# Cardiovascular Hypertensive Crisis: Recent Evidence and Review of the Literature

**DOI:** 10.3389/fcvm.2016.00051

**Published:** 2017-01-10

**Authors:** Christos Varounis, Vasiliki Katsi, Petros Nihoyannopoulos, John Lekakis, Dimitris Tousoulis

**Affiliations:** ^1^2nd Department of Cardiology, Attikon University Hospital, Athens, Greece; ^2^Cardiology Department, Hippokration General Hospital, Athens, Greece; ^3^1st Department of Cardiology, Athens University Medical School, Hippokration Hospital, Athens, Greece

**Keywords:** hypertensive crisis, management, hypertensive emergencies, hypertensive urgencies, hypertension

## Abstract

Despite the high prevalence of hypertension (HTN), only a small proportion of the hypertensive patients will ultimately develop hypertensive crisis. In fact, some patients with hypertensive crisis do not report a history of HTN or previous use of antihypertensive medication. The majority of the patients with hypertensive crisis often report non-specific symptoms, whereas heart-related symptoms (dyspnea, chest pain, arrhythmias, and syncope) are less common. Hypertensive crises can be divided into hypertensive emergencies or hypertensive urgencies according to the presence or absence of acute target organ damage, respectively. This differentiation is an extremely useful classification in clinical practice since a different management is needed, which in turn has a significant effect on the morbidity and mortality of these patients. Therefore, it is very crucial for the physician in the emergency department to identify the hypertensive emergencies and to manage them through blood pressure lowering medications in order to avoid further target organ damage or deterioration. The aim of this narrative review is to summarize the recent evidence in an effort to improve the awareness, recognition, risk stratification, and treatment of hypertensive crisis in patients referred to the emergency department.

## Introduction

Systemic hypertension (HTN) is the most common chronic medical disorder affecting over 1 billion people worldwide and more than 65 million adults in the Unites States (1). Worldwide, recent estimations indicate that HTN is the responsible cause for approximately 7.1 million deaths per year (2). In addition to this, HTN is one of the most frequent causes of visit to the physician’s office (3). Among the HTN population, about 1–2% of the patients will ultimately develop hypertensive crisis, which according to the 2003 Joint National Committee (JNC) on Prevention, Detection, Evaluation, and Treatment of High Blood Pressure (JNC 7) is defined as the elevation of systolic blood pressure (SBP) >179 mmHg or diastolic blood pressure (DBP) >109 mmHg (4). Hypertensive crises can be divided further into hypertensive emergencies or hypertensive urgencies according to the presence or absence of acute target organ damage, respectively. Target organ damage can be defined as the acute damage and resulting dysfunction of the eyes (fundoscopy findings, such as hemorrhages, exudates, or papilledema), the brain (hypertensive encephalopathy), the heart (acute pulmonary edema), or the kidneys (acute renal failure). This differentiation is an extremely useful entity in clinical practice given that a different management is needed, which in turn has significant effect on the morbidity and mortality of these patients. More specifically, in hypertensive urgencies, the blood pressure (BP) should be reduced within 24–48 h, whereas hypertensive emergencies require immediate BP reduction in order to prevent irreversible target organ damage (2). However, despite this distinction, a patient presenting with hypertensive urgency may have history of previous end-organ damage and chronic HTN without ongoing or imminent target organ dysfunction (2, 5).

The aim of this narrative review is to summarize the recent evidence in an effort to improve the awareness, recognition, risk stratification, and treatment of hypertensive crisis in patients referred to the emergency department.

## Epidemiology and Clinical Profile

In a recent large multicenter Italian study (6), 4.6/1,000 cases—out of 333,407 patients—consecutively admitted to emergency department were diagnosed with hypertensive crises (*n* = 1,546). Out of 1,546 hypertensive cases, 25.3% of them (*n* = 391) being reported as hypertensive emergencies. Interestingly, 23% of the emergencies occurred in patients with unknown HTN (27.9% among men and 18.5% among women). Regarding symptoms, the majority (55.6%) of the hypertensive crisis patients reported non-specific symptoms like headache without neurological deficit, dizziness, vomits, palpitations, etc., even among emergency cases (49.3%). Moreover, heart-related symptoms (dyspnea, chest pain, arrhythmias, and syncope) were the less common symptoms in hypertensive crises (28.3%). Regarding hypertensive emergencies, the majority (30.9%) of the patients had acute pulmonary edema, 22% had stroke, and 17.9% had myocardial infarction. Less frequent diagnoses were acute aortic dissection (7.9%), acute renal failure, and hypertensive encephalopathy (4.9%). Also, patients with hypertensive emergencies had 34% higher odds of being male and 28% less odds of having non-specific symptoms compared with patients with hypertensive urgencies. The importance of this study was that the frequency of unknown HTN both in the hypertensive crises and more specifically in the hypertensive emergencies was higher compared to previous studies published in the literature.

Regarding the clinical profile of the patients presenting with hypertensive crises, a recent cross-sectional study in Brazil (7) revealed that about 88% of the patients with hypertensive crisis reported known history of HTN, whereas approximately 76% of the patients were treated previously with antihypertensive treatment. Patients presenting with hypertensive emergency were older (63.4 ± 13.4 vs. 57.0 ± 15.6, *p* < 0.001), more sedentary, and antihypertensive medication were less frequently prescribed compared to those with hypertensive urgency, whereas no other differences were found in terms of other cardiovascular risk factors, like smoking or prevalence of diabetes mellitus.

Different symptoms and clinical profile have been reported in studies comparing hypertensive emergencies and urgencies. More specifically, age and diastolic pressure were higher in hypertensive emergencies than hypertensive urgencies. The most frequent sign and symptom in hypertensive urgency was headache (22%) and chest pain (27%) followed by dyspnea (22%) in hypertensive emergencies. End-organ damage in hypertensive emergencies was associated more frequently with cerebral infarction (24%), acute pulmonary edema (23%), and hypertensive encephalopathy (16%) (8). Similarly, in a recent study of 73,063 hypertensive patients presenting to the emergency room from 2005 to 2010, patients with hypertensive crisis were more frequently presented with headache and chest pain (9).

## Pathophysiology

The precise pathophysiology of the hypertensive crisis remains unclear. However, two different but interrelated mechanisms may play a central role in the pathophysiology of the hypertensive crisis. The first is the failure in autoregulatory mechanism in the vascular bed. The autoregulation system is a key factor in the pathophysiology of HTN and hypertensive crisis. Autoregulation is defined as the ability of the organs (brain, heart, and kidneys) to maintain a stable blood flow irrespective of alterations of perfusion pressure. If the perfusion pressure drops, the corresponding blood flow decreases temporarily, but it returns to normal values after the next few minutes. In case of autoregulation malfunction, if the perfusion pressure drops, this leads to decrease in blood flow and an increase in vascular resistance. In hypertensive crisis, there is a lack of autoregulation in vascular bed and blood flow and so an abrupt increase of BP and systemic vascular resistance can occur, which often leads to mechanical stress and endothelial injury (10).

**Figure 1:**
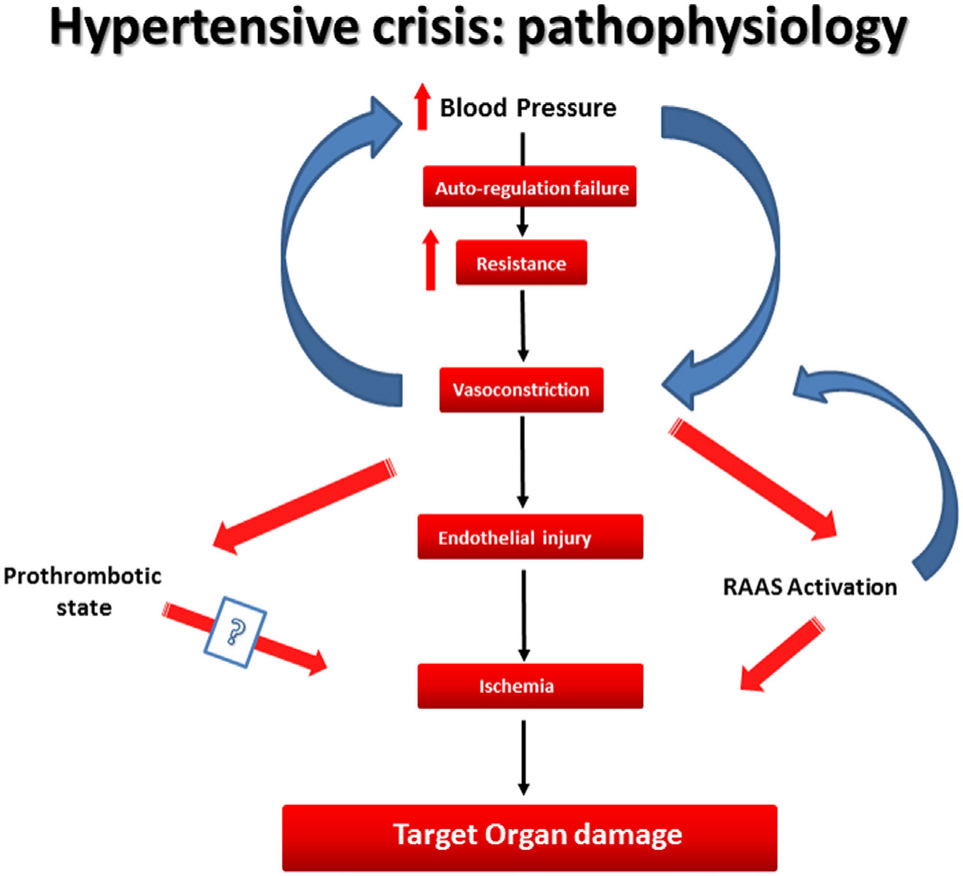
**The pathophysiology of hypertensive crisis**.

The second mechanism is the activation of renin–angiotensin system, leading to further vasoconstriction and thus generating a vicious cycle of continuous injury and subsequently ischemia (2). Besides these mechanisms, a prothrombotic state may play a key role in hypertensive crisis; a recent, albeit small, study showed that sP-selectin was significantly higher in patients with hypertensive crisis compared with normotensive controls regardless of the presence of retinopathy, which suggests that platelet activation is a relatively early finding in the pathophysiologic sequelae of hypertensive crisis (11) (Figure 1).

## Diagnosis

The evaluation of a hypertensive crisis initially includes a detailed medical history. More specifically, the physician should ask the patient about (i) the duration of HTN history, (ii) any evidence of uncontrolled BP recordings in the past, (iii) concomitant administration of other drugs that might increase BP (e.g., non-steroidal anti-inflammatory drugs), (iv) history of sleep apnea syndrome, and (v) evaluation of cardiovascular risk factors and other comorbidities.

Also, physical examination should include (i) auscultation of heart sounds/murmurs (aortic coarctation), neck arteries, and abdominal murmurs, (ii) neurological deficits, (iii) fundoscopy to assess for retinopathy, grade III (flame hemorrhages, dot and blot hemorrhages, hard and soft exudates), or grade IV (papilledema), (iv) absence, reduction, or asymmetry of pulses in the lower extremities, and (v) examination of the abdomen (aortic aneurysm). Also, vital signs should be checked carefully in the evaluation of a patient with hypertensive crisis, e.g., BP, oxygen saturation, and heart rate. More specifically, BP should be measured in both the arms to detect any potential differences (2). In a recent study of 189 patients, normal heart rate was associated with hypertensive urgency, whereas patients with hypertensive emergency had higher average heart rate. Tachycardia was most associated with hypertensive left ventricular failure in the setting of hypertensive emergency due to activation of sympathetic nervous system. Moreover, heart rate less than 100 bpm had high specificity, classifying patients as hypertensive urgencies. However, other hemodynamic parameters such as systolic or DBP and pulse pressure were not able to classify hypertensive emergencies from hypertensive urgencies (12). This vital sign seems to be useful in the emergency department in the differentiation of hypertensive emergencies from hypertensive urgencies.

Initial laboratory analyses should be performed rapidly after initial evaluation of the patient. These laboratory analyses include urinalysis (check for significant proteinuria, red blood cells, cellular casts, or analysis for metanephrines in case of high suspicion of pheochromocytoma), a chemistry panel (creatinine, blood urea nitrogen levels), electrocardiogram (to check for myocardial ischemia or infarction and/or signs of left ventricular hypertrophy), a plain chest radiograph (evaluation of cardiomegaly or pulmonary edema), brain computed tomography (CT) scan (evaluation of neurological deficits), and chest CT scan or transesophageal echocardiography (in suspicion of aortic dissection) (2).

A fast transthoracic echocardiogram could be also useful to assess the function of the left ventricle to measure the ejection fraction or to detect segmental hypokinesias. Even if new echocardiography imaging techniques are not used extensively in daily clinical practice, it seems that speckle-tracking echocardiography may detect depressions of global longitudinal left ventricular systolic strain and global systolic strain rate during hypertensive crisis as opposed to left ventricular ejection fraction (13). Despite this interesting finding, speckle tracking echocardiography is not easy to implement in an emergency department setting.

## Treatment

It seems that patients with hypertensive crises, regardless of whether it is the emergency or urgency subtype, receive treatment mostly in a rather heterogeneous and empirical way. However, important treatment options are available to emergency department physicians.

In cases with hypertensive urgency BP control should be managed with the use of low doses of oral antihypertensive medications, where a gradual decrease of BP over hours to days is expected. Medications that can be used to treat hypertensive urgencies are oral labetalol (3:1 ratio of antagonism of non-selective β-adrenergic and *a*1 receptor) and clonidine (central *a*-2 agonist).

On the other hand, hypertensive emergencies require rapid BP control with a parenteral antihypertensive medication, and in these instances the patient should be admitted to intensive care unit. The BP should be reduced within minutes to an hour to about 20–25% in the first hour and then to 160/100 or 160/110 mmHg within the next 2–6 h. However, BP should not be returned to normal values (14). This is due to the fact that additional BP reduction could cause brain ischemia due to abnormal cerebral flow autoregulation in these patients. An exception to this would be in the case of aortic dissection, where the rapid and immediate reduction of BP within 5–10 min using initially a parenteral β-blocker (i.e., esmolol) (BP target: SBP <120 mmHg and a mean arterial pressure <80 mmHg) is crucial for the patient (14, 15).

A great number of medications are available for the treatment of hypertensive emergencies. Sodium nitroprusside is a first-choice for the majority of hypertensive emergencies, and it acts within seconds as a potent arterial and venous dilator. The most important disadvantage is thiocyanate toxicity. The toxicity is more likely to occur if patients have hepatic or renal failure and when the agent is administered for more than 48–72 h (16).

Labetalol can be used to treat hypertensive emergencies through IV administration with a non-selective β-blocker and *a*1 adrenergic receptor blocker with 6.9:1 ratio of antagonism reducing the systemic vascular resistance but maintaining the cerebral, renal, and coronary blood flow. It is interesting that despite the β-blocking effect it maintains also the cardiac output (2).

Nitroglycerine is a venodilator that mainly reduces the preload and decreases the cardiac oxygen demands, and it is often used in hypertensive crises. This agent is used primarily in acute myocardial infarction and acute pulmonary edema along with other antihypertensive regimens (17).

Other agents that can be used in hypertensive emergencies include nicardipine (dihydropyridine calcium channel blocker), which is a useful agent for patients with coronary artery disease due to its beneficial effect on coronary blood flow or clevidipine, which is a new short-acting intravenous dihydropyridine calcium channel blocker (14). Enalaprilat is an angiotensin-converting enzyme inhibitor, but it is not recommended since it can aggravate renal blood flow, and the potential of renal failure in patients with hypertensive emergency is high (18, 19). Fenoldopam is an important medication, and it acts through peripheral dopamine-1 receptors as a vasodilator and as a diuretic. It has been shown to be an effective and well-tolerated agent for the treatment of hypertensive emergency (20). Diuretics are not generally recommended agents for the treatment of hypertensive emergencies with the exception of acute pulmonary edema. However, in a recent small randomized controlled trial (21), researchers randomized 59 patients with acute pulmonary edema due to hypertensive crisis to either furosemide or placebo. Researchers concluded that the subjective perception of dyspnea in patients with hypertensive pulmonary edema was not influenced by the administration of a loop-diuretic. This may be due to the fact that patients with hypertensive heart failure are often euvolemic or only mildly hypervolemic and also that loop-diuretics and especially furosemide after IV administration initially exert vasodilation of the venous capacitance.

## Prognosis

It seems that hypertensive emergencies have different prognoses compared to hypertensive urgencies. Due to the fact that several medications can be used to treat hypertensive crises, epidemiological data show that the mortality of the hypertensive emergency has been decreased gradually from 80% in 1928 to 10% in 1989 (22). In a recent study with hypertensive crisis patients who were admitted to a coronary care unit, researchers found that the overall mortality was 3.7%. In patients with hypertensive emergency, the mortality was higher (4.6%) compared with patients with hypertensive urgencies (0.8%) (23). Despite the different prognosis between patients with two entities, other researchers tried to find prognostic factors of major adverse cardiac or cerebrovascular events (MACCE) defined as a composite of myocardial infarction, unstable angina, hypertensive crisis, pulmonary edema, stroke, or transient ischemic attack. In a recent retrospective study with a 2-year duration, patients with hypertensive crises and elevated cardiac troponin-I (c TnI) had 2.7 times higher risk for the occurrence of MACCE at 2-years follow-up compared with those with normal c TnI values (24). The introduction of a prognostic score from large epidemiological studies could be of high value in order to stratify the patients according to baseline clinical and demographic characteristics.

## Conclusion

Hypertensive crisis has the potential of end-organ damage, and this has a significant effect on patient’s prognosis. The prognosis differs substantially whether the patient is presenting with hypertensive emergency or urgency. Several regimens are effective to treat both hypertensive emergencies and urgencies, but the choice of the treatment is dependent on the clinical presentation of the patient. It is very crucial for the physician in the emergency department to identify the hypertensive emergencies quickly and so to intervene with BP-lowering medications in order to avoid further target organ damage and deterioration.

## Author Contributions

CV, VK, PN, JL, and DT declare the following criteria regarding the submitted work: (1) substantial contribution to the conception or design of the work; (2) drafting the work or revising it critically for important intellectual content; (3) final approval of the version to be published; and (4) agreement to be accountable for all aspects of the work in ensuring that questions related to the accuracy or integrity of any part of the work are appropriately investigated and resolved.

## Conflict of Interest Statement

The authors declare that the research was conducted in the absence of any commercial or financial relationships that could be construed as a potential conflict of interest.
